# Supplementary material for: Tumor-reprogrammed resident T cells resist radiation to control tumors
Source: Nat Commun. 2019 Sep 2;10:3959. doi: 10.1038/s41467-019-11906-2 (PMC6718618; doi:10.1038/s41467-019-11906-2)
Supplement: Supplementary file 1 — Supplementary Information [file 41467_2019_11906_MOESM1_ESM.pdf]

Supplementary Information

Tumor-reprogrammed resident T cell resist radiation to control tumors

Arina et al.

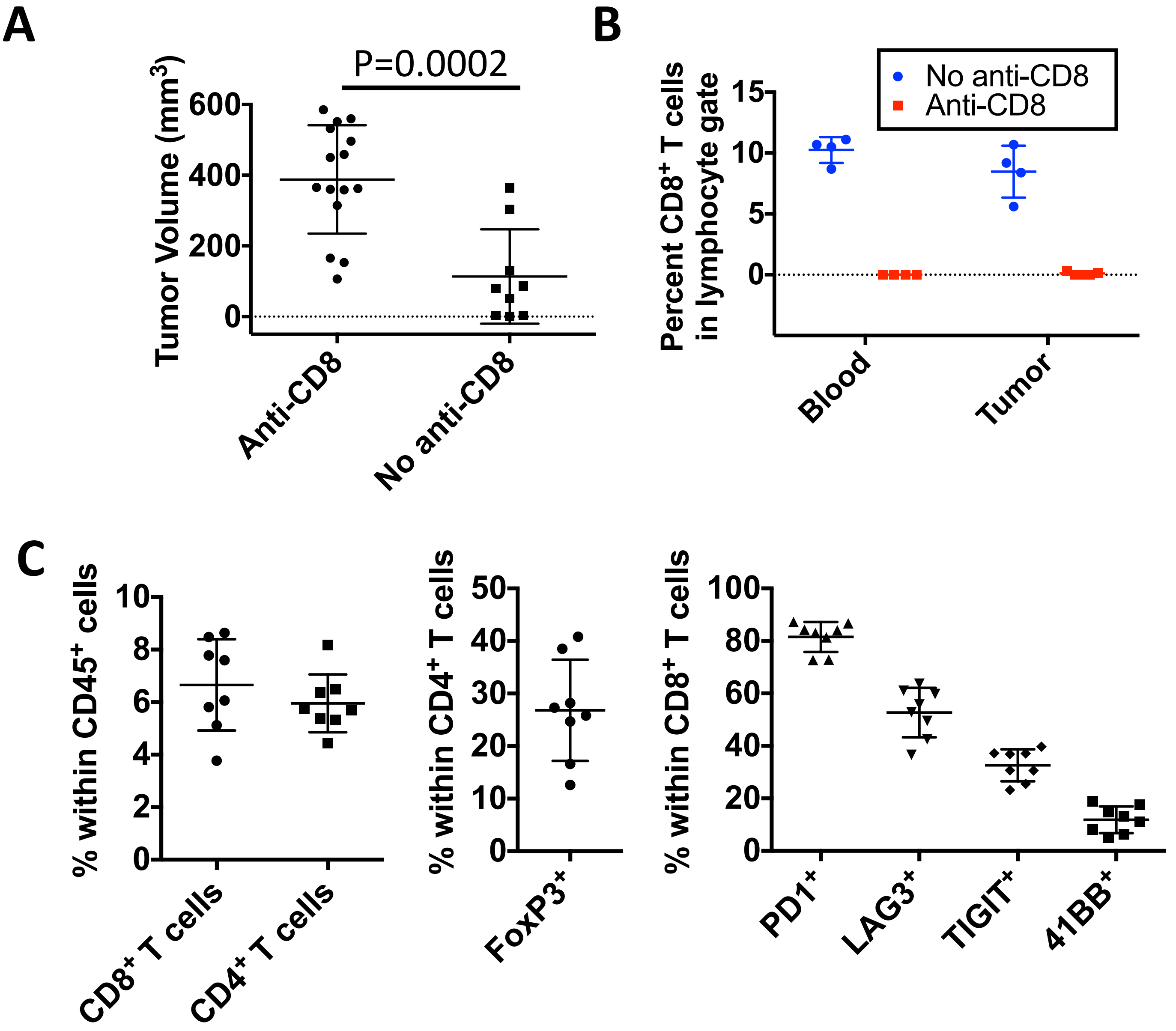

Supplementary Fig. 1. A single dose of depleting anti-CD8 antibodies at the time of tumor injection promotes Panc02SIYCerulean tumor growth but allows the development of an intratumoral T cell infiltrate with an exhausted phenotype by the time of treatment with IR. A. B6 mice were injected with $5x10^6$ Panc02SIYCerulean. Mice received or not a single 200 µg dose of anti-CD8 depleting antibodies (10F.9G2, BioXCell) on the day of tumor inoculation. Shown tumor size at day 27-29 since tumor cell inoculation (unpaired t-test). Data were pooled from 4 individual experiments. **B.** Anti-CD8 antibody depletes tumor-infiltrating T cells in addition to circulating T cells. Levels of $CD8^+$ T cells in the blood and tumors of mice bearing established MC38 tumors and treated 24 h before sacrifice with a single 200 ug dose of anti-CD8 antibodies. Control mice were untreated. 4 mice per condition. **C.** Characterization of the Panc02SIYCerulean intratumoral T cell phenotype at the time mice are treated with IR (3 weeks). Mice were injected with $5x10^6$ Panc02SIYCerulean and a single 200 µg dose of anti-CD8 at day 0. Each dot is an individual mouse. Data are from one experiment with 8 mice. All plots show average and S.D.

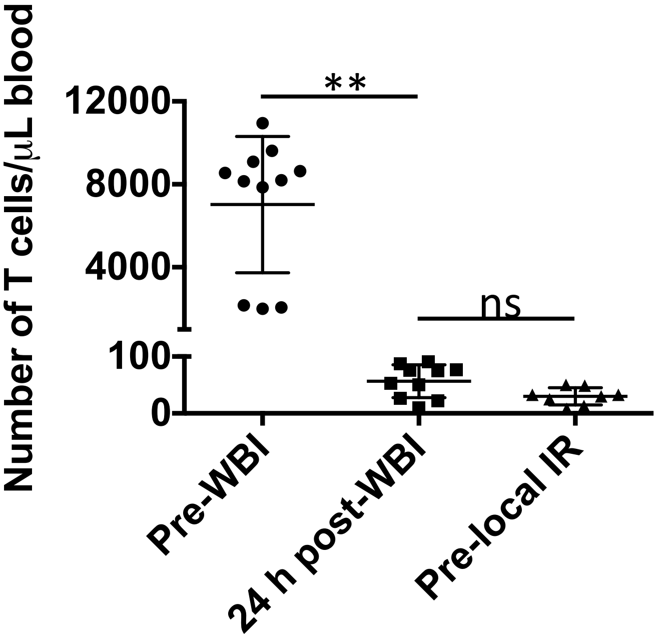

Supplementary Fig. 2. Circulating EYFP+ T cells are significantly decreased after 8 Gy WBI and remain at low levels at the time of treatment with local IR . Panc02SIYCerulean cancer cells were injected s.c. into T cell reporter mice. On day 21, mice received 8 Gy WBI while tumors were shielded as in Figure 1. The number of EYFP+ cells was measured in the peripheral blood before WBI, 24 h after WBI, and immediately before tumor treatment with local IR (day 5 after WBI). N=8-11 mice, shown average and S.D. **, P=002; ns, non-significant, Wilcoxon test) . An experiment is shown from 2 independent experiments with similar results.

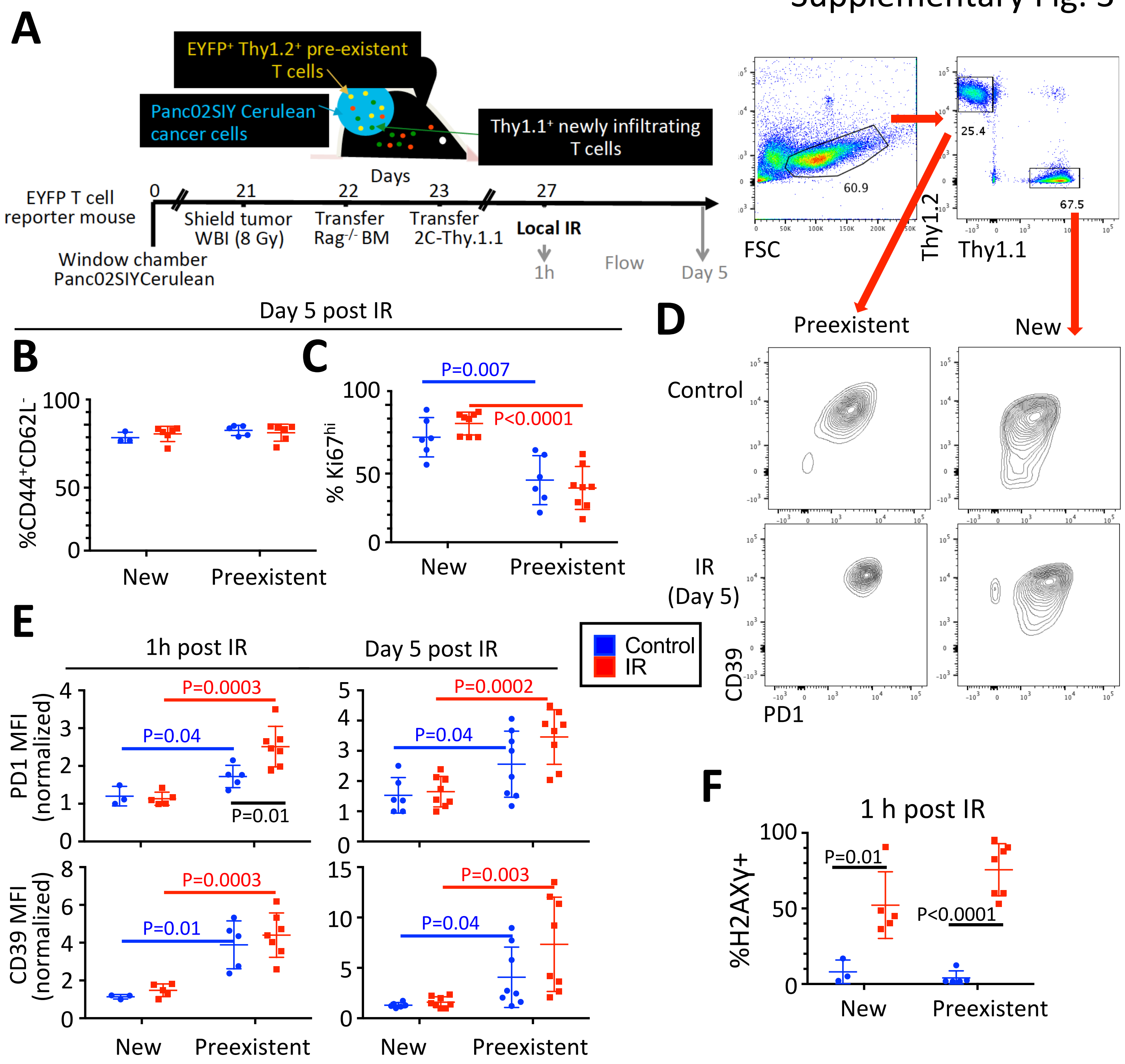

Supplementary Fig. 3. Phenotypes of preexistent and newly infiltrating T cells in tumors treated or not with IR. A. Experimental scheme and gating strategy for preexistent and newly infiltrating T cells. Experimental design is the same as used in Figure 1, but here 2C-Thy1.1⁺ were used as "newly infiltrating" T cells instead of 2C-EGFP⁺ T cells. Mice were sacrificed 1 h or 5 days after local IR and the tumors analyzed by flow cytometry. Gating strategy on magnetically enriched CD8⁺ T cells purified from tumors. **B**. The majority of both preexistent and newly infiltrating T cells are CD44⁺CD62L⁻ **C.** Preexistent T cells express lower levels of ki67 antigen compared to newly infiltrating T cells. **D-E.** Preexistent T cells express higher levels of exhaustion-associated markers PD1 and CD39 than newly infitrating T cells (**D**, representative examples; **E**, quantification) **F.** 1 h after local IR, T cells in irradiated tumors show evidence of DNA damage, as increased percentages of phosphorylated H2AXγ. Data are pooled from 2 independent experiments for 1 h after IR and another 2 independent experiments for Day 5, with a total N=5-7 (1h) and N=6-8 (Day 5) per group. Data in E were normalized to the minimum expression value within each experiment before pooling the data. Plots in B, C, E and F show average and S.D. and were analyzed using unpaired t-tests.

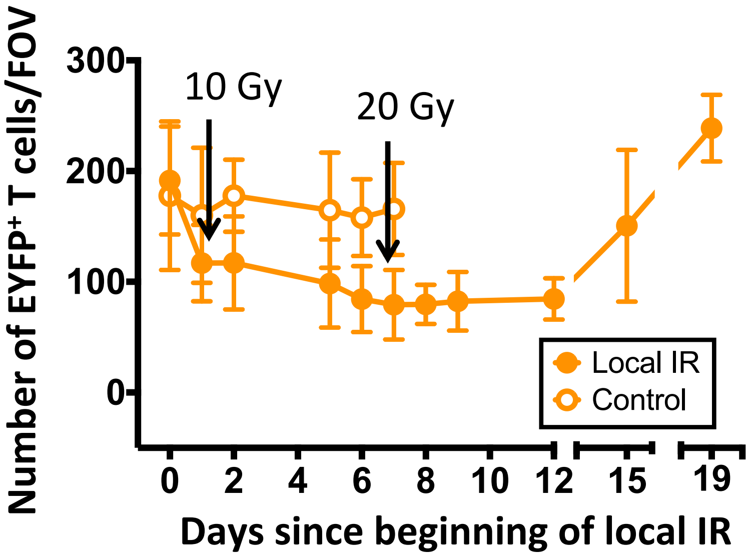

Sup. Figure 4. Survival of preexistent intratumoral T cells in MC38 tumors after 30 Gy local IR. MC38Cerulean cancer cells were injected s.c. into two T cell reporter mice (Lck-EYFP) bearing dorsal window chambers. When tumors were established (day 14), mice received a myeloablative dose (8 Gy) of WBI while their tumors were shielded, and bone-marrow was reconstituted by injection of DsRed^{+}Rag$^{-/-}$ cells within 24 h. 5 days after BM cell transfer, one mouse received 10 Gy followed by 20 Gy 4 days later as indicated in the figure. The control mouse received no local IR. Longitudinal in vivo imaging was performed on the mice before, during and after IR. Numbers of EYFP^{+} T cells were determined over time in 9-13 optical regions per mouse (averages and S.D. shown).

Supplementary Fig. 5

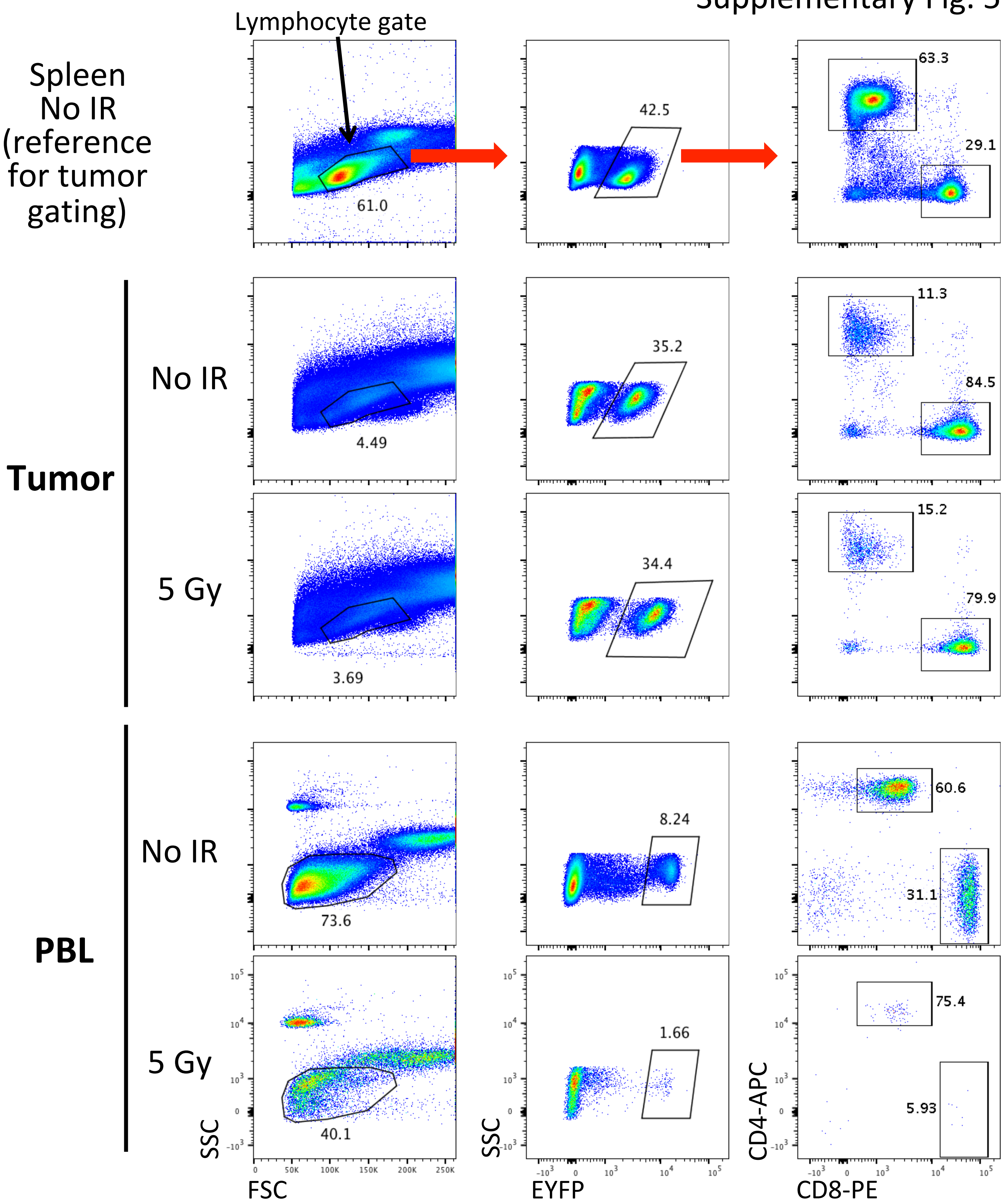

Supplementary Fig. 5. Gating strategy for determination of percentages of circulating and intratumoral T cells in T cell reporter mice receiving WBI (related to Figures 3A and 3B). Gates were first drawn on a spleen sample stained in parallel (top) and then copied into tumor sample plots. Gates for PBL samples were drawn independently since samples were processed in a different fashion as detailed in Materials and Methods. The % EYFP^{+} (T) cells were determined in the lymphocyte gates as drawn above. Then the distribution of EYFP^{+} T cells in CD4^{+} and CD8+ T cells was analyzed as indicated.

Supplementary Fig. 6

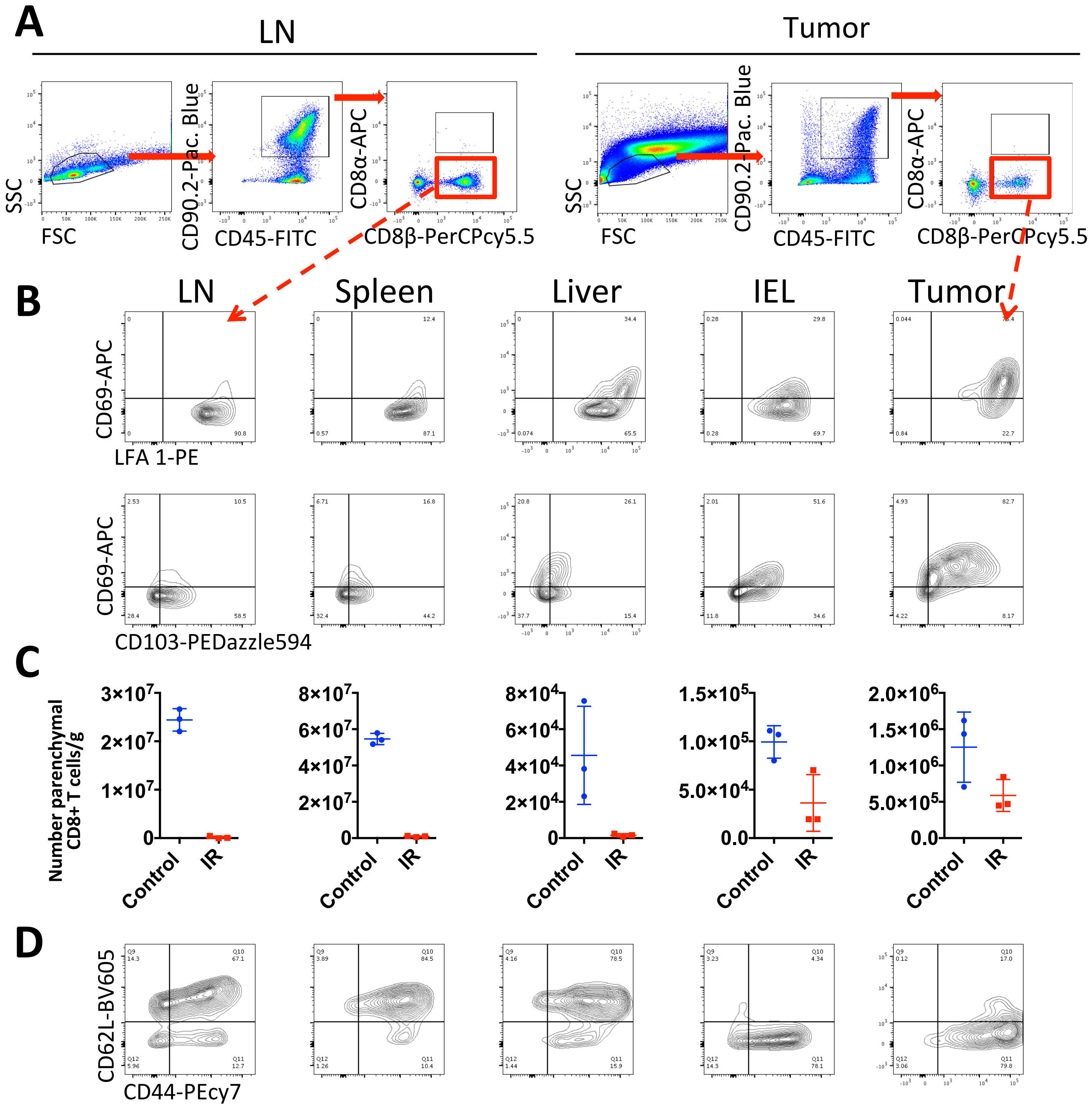

Supplementary Fig. 6 (related to Figure 3). Phenotype of parenchymal CD8⁺ T cells in MC38 tumor-bearing mice. B6 mice bearing established MC38 tumors were injected with anti-CD8α-APC i.v. Five minutes later, they were sacrificed and perfused with PBS, and their organs were extracted. **A.** Gating strategy for parenchymal CD8⁺ T cells is shown in representative examples of LN and tumor; the same strategy was used for all tissues/organs. **B.** Flow cytometry plots showing expression of markers associated with a T_{RM} phenotype by parenchymal CD8⁺ T cells from different organs/tissues. **C.** Absolute numbers of parenchymal CD8⁺ T cells in several organs before/after 8 Gy WBI (average and S.D.). **D.** Expression of markers associated with a memory phenotype by parenchymal CD8⁺ T cells. Data are from one experiment with 3 mice per group, representative of two experiments performed with similar results. Samples used for this figure are the same as used in main Fig. 3C-F.

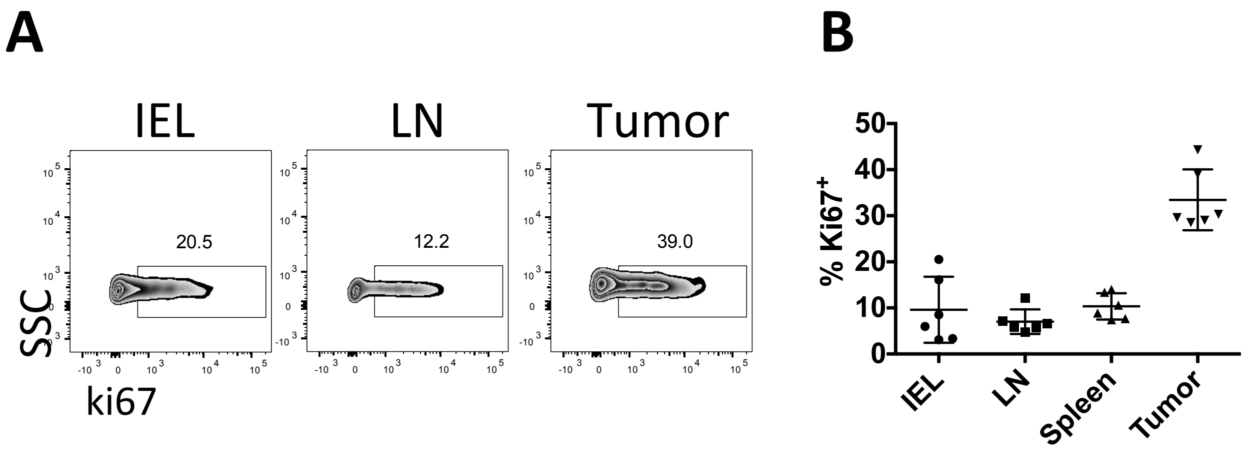

Supplementary Fig. 7. **Ki67 expression levels in radiosensitive and radio-resistant T cells.** Single cell suspensions from the indicated organs were analyzed by flow cytometry to determine the % of $Ki67^{+}$ cells in the $CD90.2^{+}CD8\beta PerCPcy5.5^{+}CD8\alpha APC^{-}$ (parenchymal $CD8^{+}$ T cell) population. N=6 mice. A. Representative examples for IEL, LN and tumor, B. Data summary, showing average and S.D.

Supplementary Fig. 8

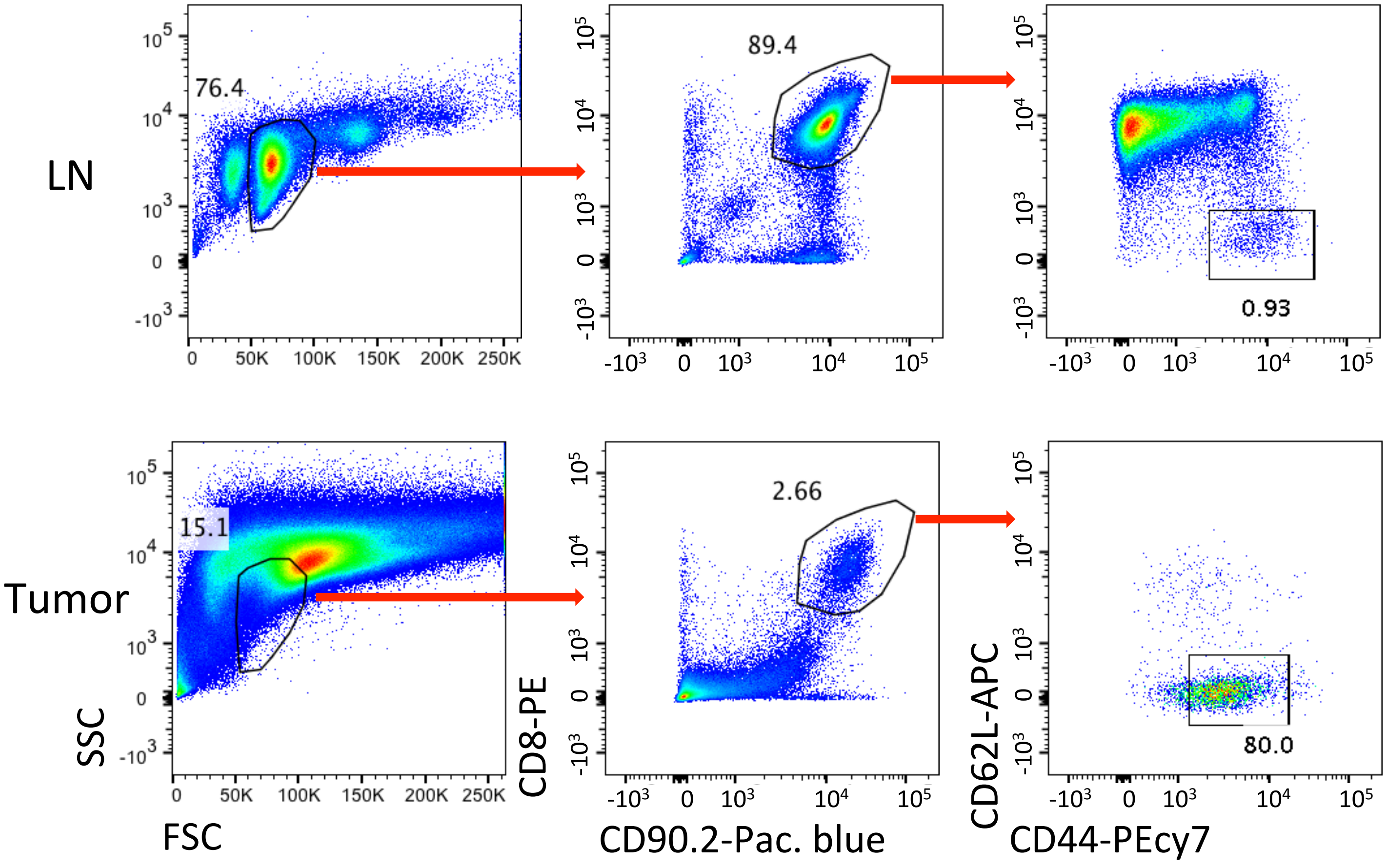

Supplementary Fig. 8 (related to Fig. 4). Sorting gates used for isolation of $CD44^{+}CD62L^{-}$ $CD8^{+}$ T cells from lymph nodes and tumor of MC38 tumor-bearing mice. A representative example is shown from non-irradiated mice in one out of 2 independent experiments.

A

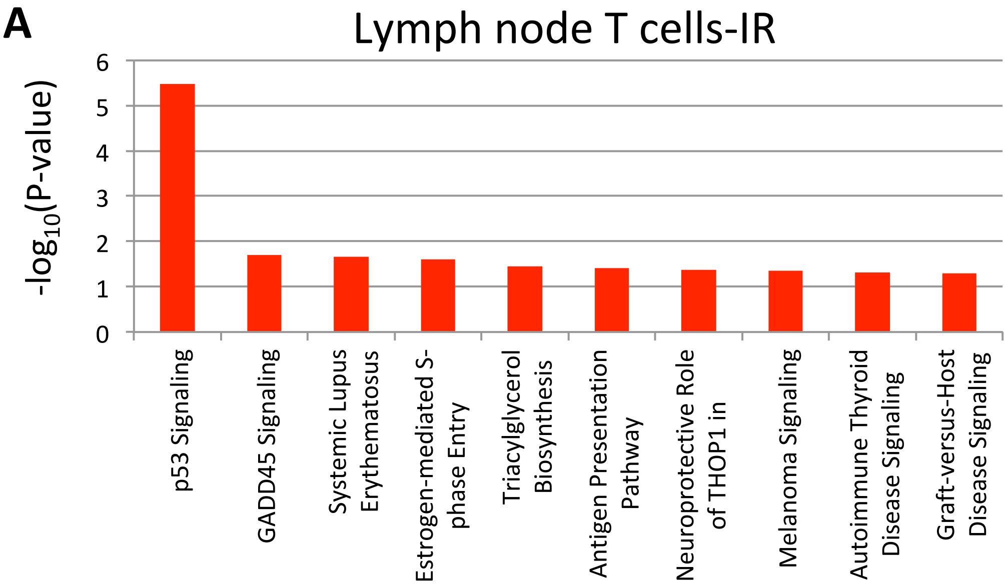

B

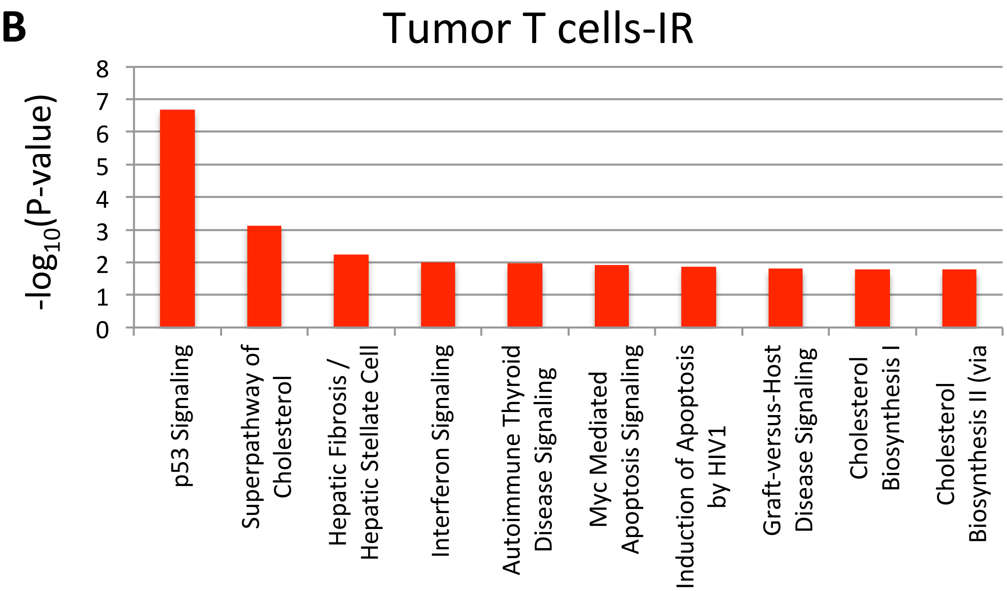

Supplementary Fig. 9. IR induces predominantly p53-signaling-related changes both in LN and tumor-infiltrating $CD44^{+}CD62L^{-}$ T cells. Top-ranked canonical pathways activated by IR in LN- and tumor-infiltrating CD44+CD62L- T cells, respectively, as determined by IPA analysis. P-value indicates enrichment for specific canonical pathway.

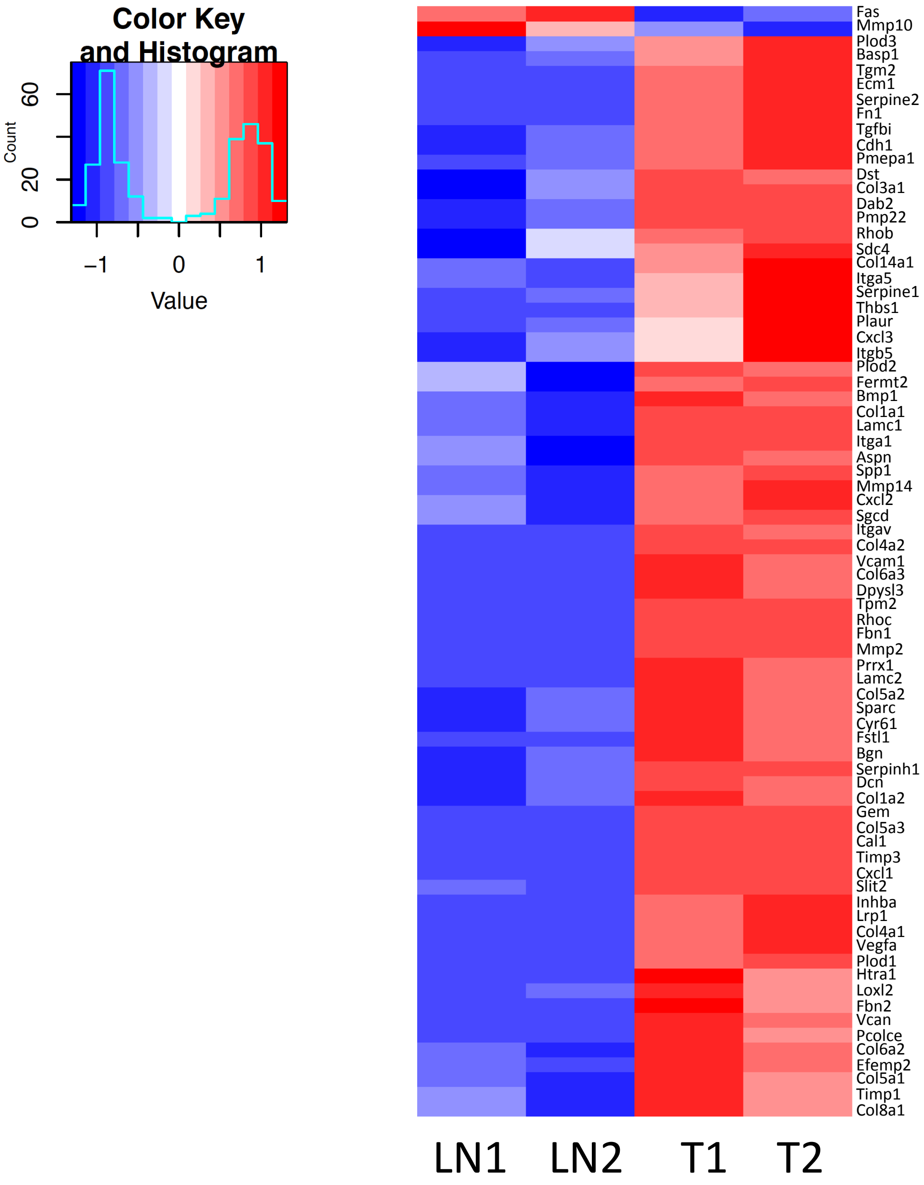

Supplementary Fig. 10. Genes from epithelial to mesenchymal transition hallmark signature (GSEA) that are differentially expressed in tumor vs. lymph node T cells. Heat map. Each array sample corresponds to pooled sorted $CD8^+CD44^+CD62L^-$ cells from inguinal and axillary LN (LN) or tumors (T) from 4 mice, in two independent experiments, generating 4 samples (LN1, LN2, T1, T2).

A

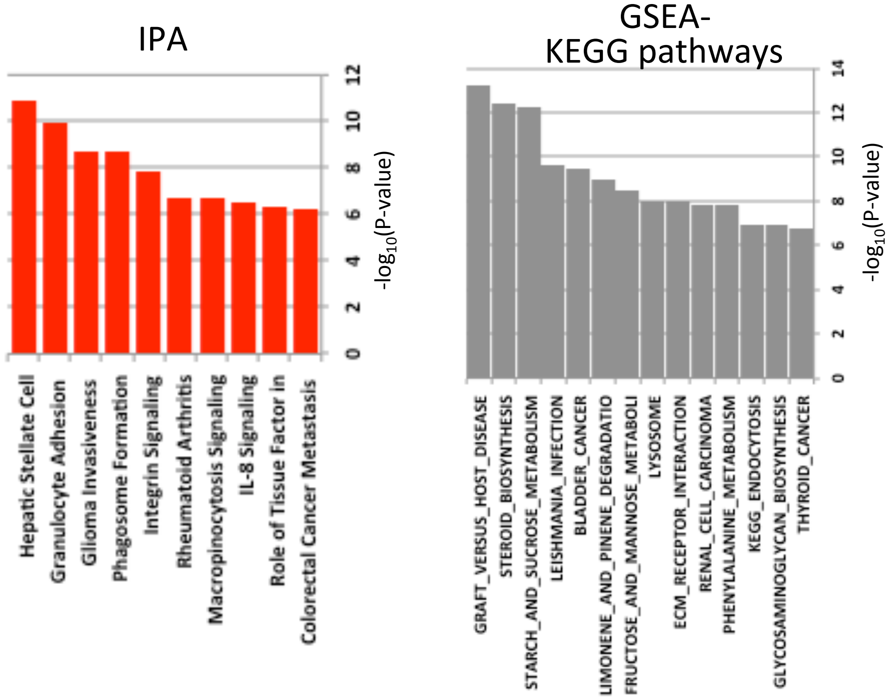

B

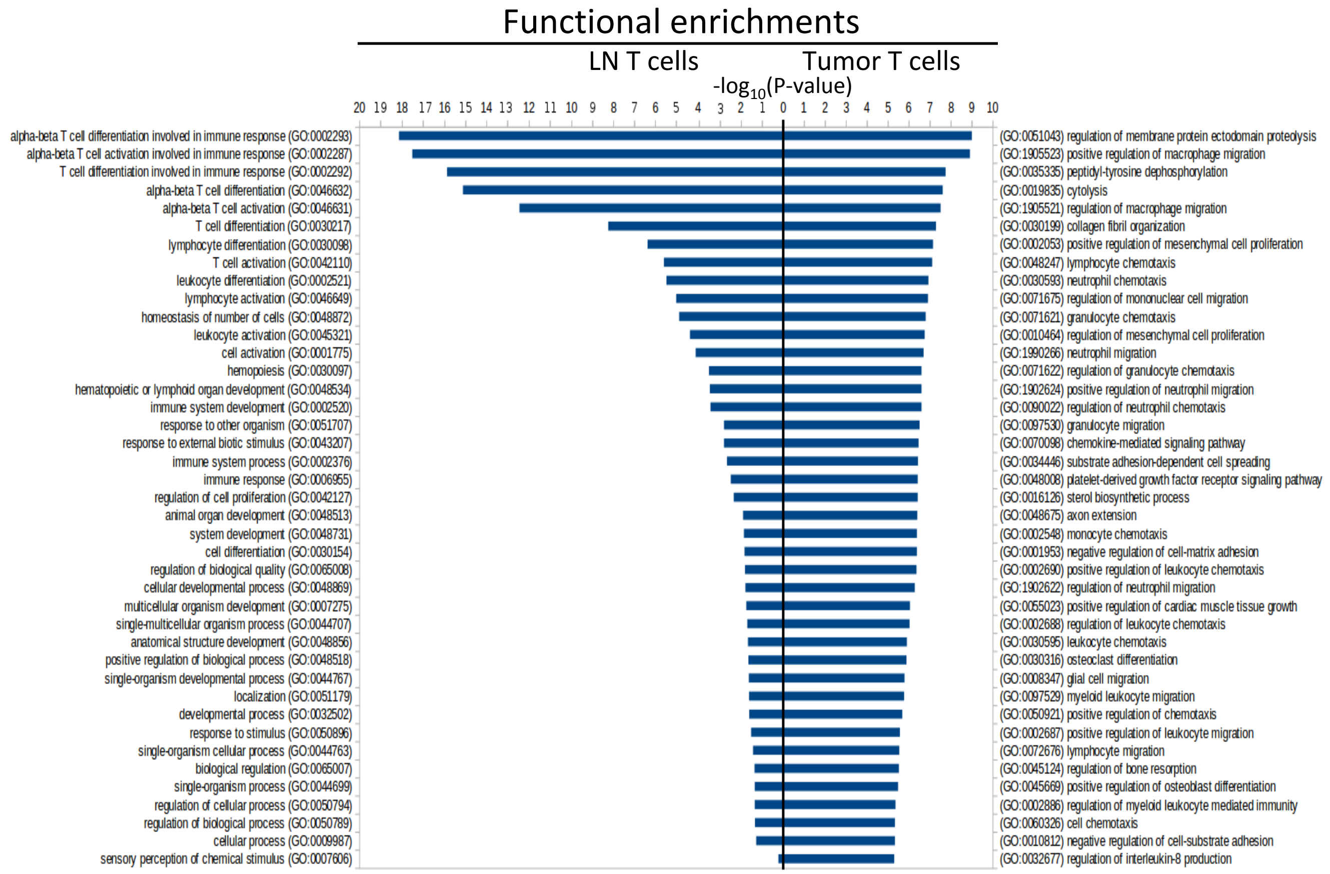

Supplementary Fig. 11. Intratumoral T cells show an activation of molecular pathways reflecting the tumor microenvironment in which they reside, and a reduction of T cell activation/differentiation-related gene expression. For Panel A, genes differentially expressed between LN and tumor T cells were analyzed using IPA (left) and GSEA (right). In Panel B, enrichment of Gene Ontology (GO) terms for genes over-expressed in LN T cells (left) and tumor T cells (right) are shown. P-value indicates enrichment for pathways or GO terms.

Supplementary Fig. 12

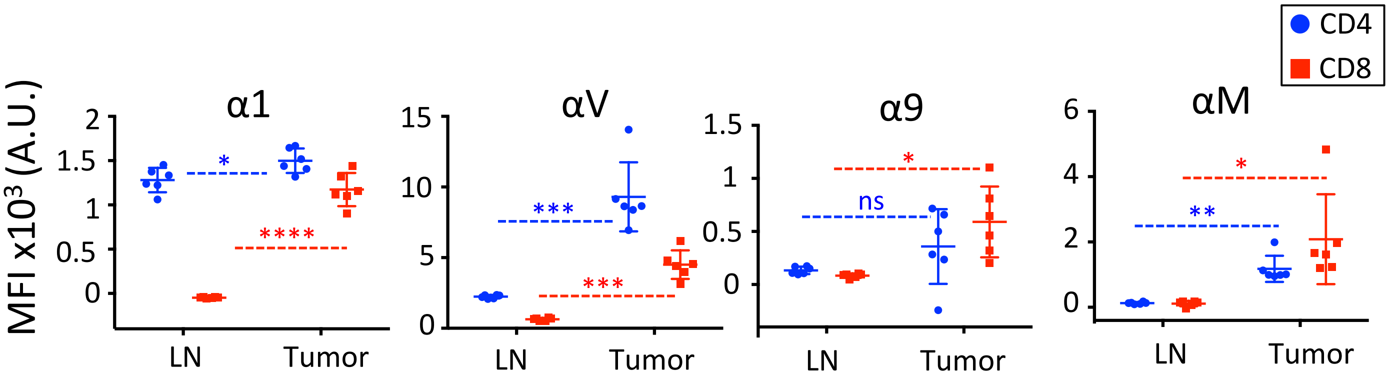

Supplementary Fig. 12. Validation of integrin subunit upregulation in intratumoral T cells at the protein level. Flow cytometry analysis of integrin expression by T cells in the tumor and LN from mice with established MC38 tumors (N=6 mice). Data are representative from 3 independent experiments and show average and S.D. *, $P \leq 0.05$; ** $P \leq 0.01$; ***, $P \leq 0.001$; ****, $P \leq 0.0001$, ns, non-significant (unpaired t-test).

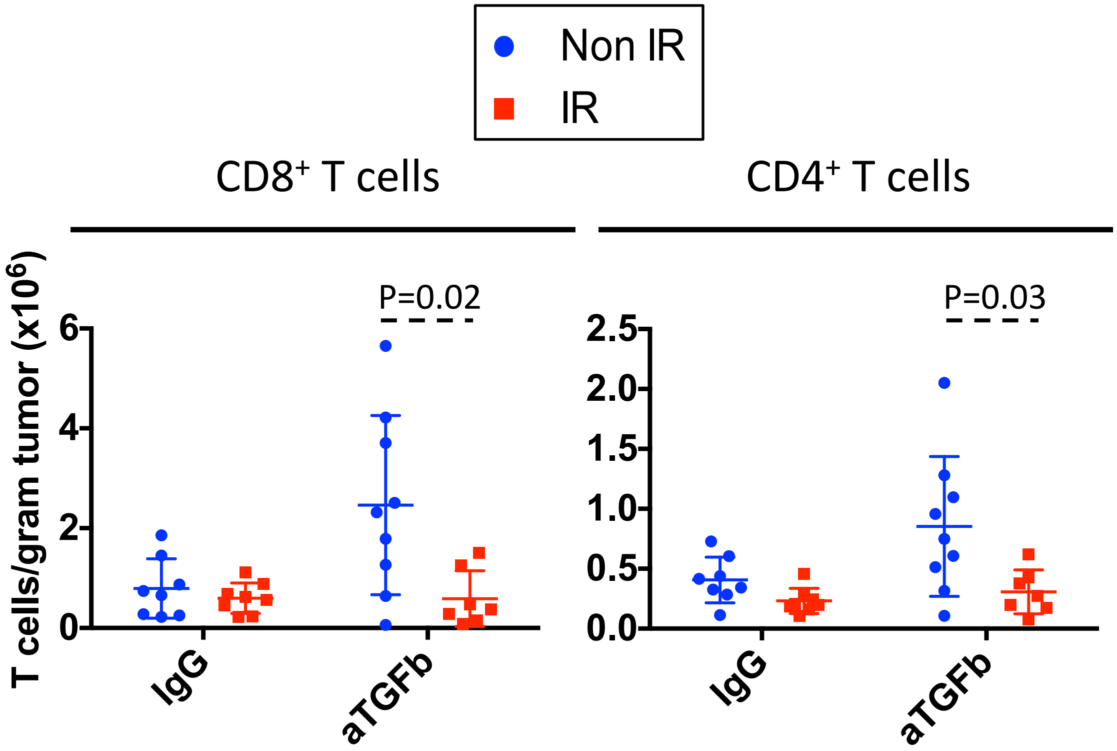

Supplementary Fig. 13. The increased effect of IR in the number of intratumoral T cells after TGFβ blockade affects CD4⁺ and CD8⁺ T cells similarly . Distribution of CD8⁺ and CD4⁺ T cells in the tumors of irradiated mice treated with TGFβ-blocking antibodies (aTGFb) or isotype control antibodies (IgG). Data correspond to the experiments shown in Fig. 5C and show average and S.D. , P values by unpaired t-test.

Supplementary Fig. 14

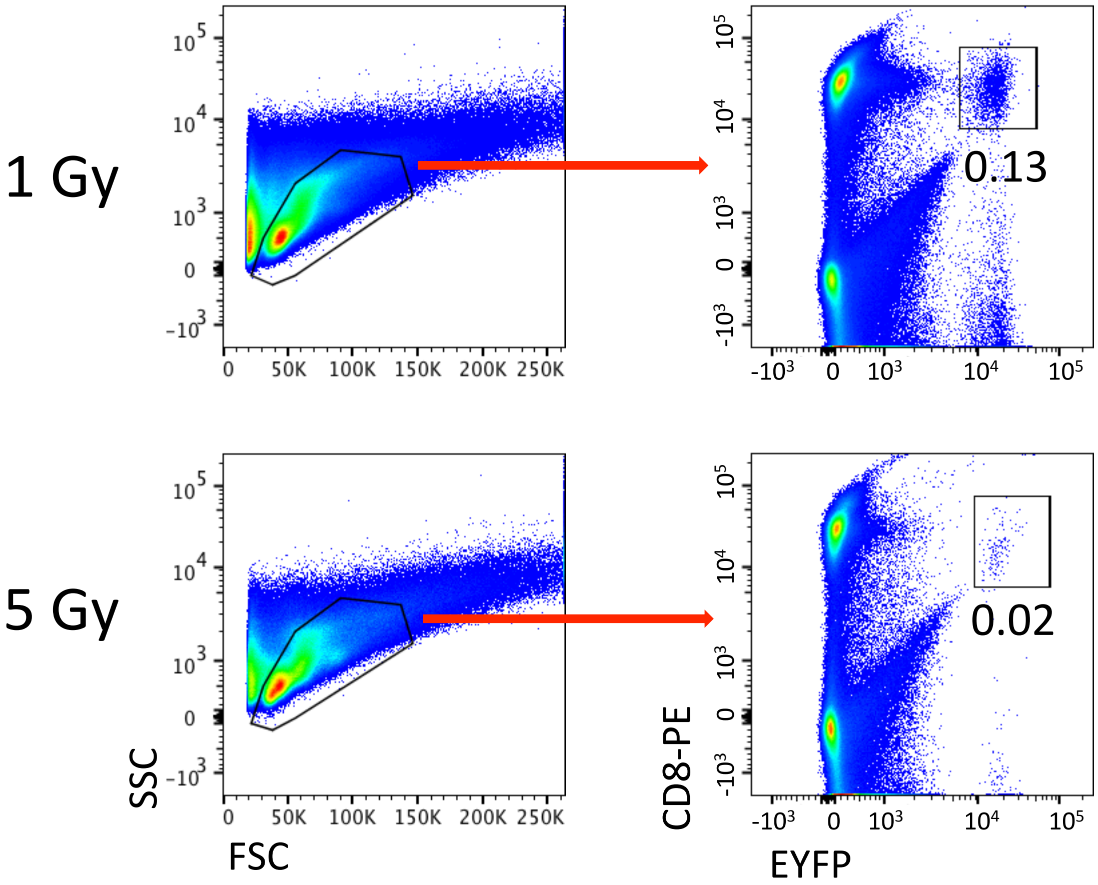

Supplementary Fig. 14 (related to Fig. 6A). Gating strategy used to quantify the levels of $CD8^{+}EYFP^{+}$ T cells present in recipient mice of tumor fragments from mice treated with different doses of WBI. Representative examples show splenocytes from $OT1Rag^{-/-}$ mice that were inoculated with MC38 tumor fragments grown in $EYFP^{+}$ T cell reporter mice treated with the indicated doses of WBI. $OT1Rag^{-/-}$ mice were sacrificed 3 weeks after tumor fragment inoculation.

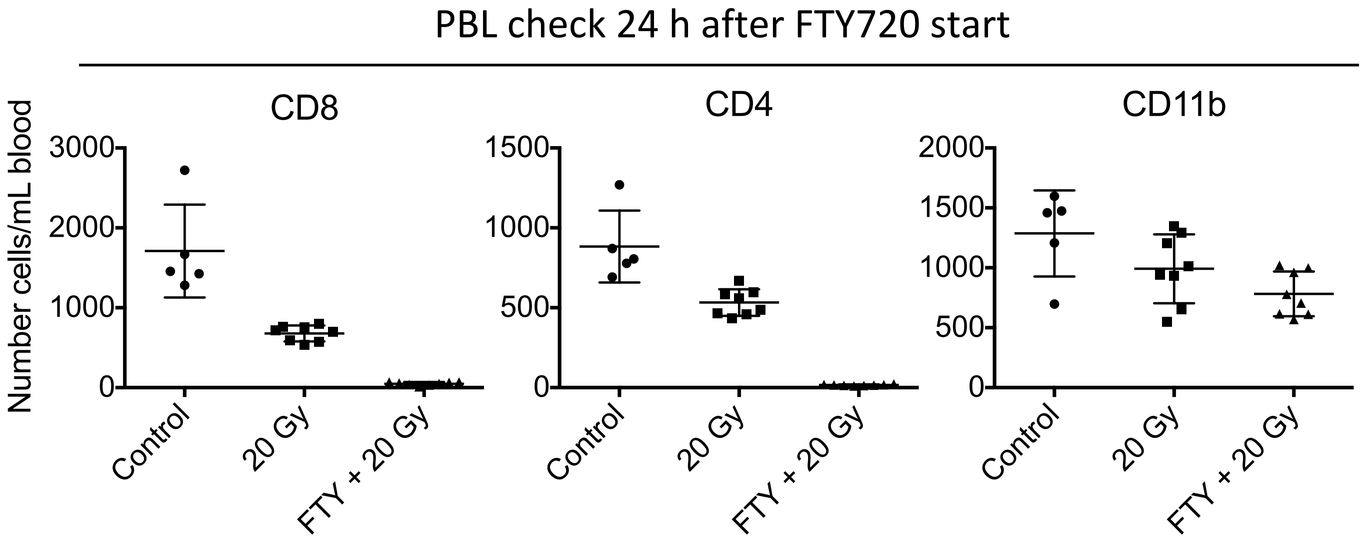

Supplementary Fig. 15. FTY720 treatment effectively eliminates circulating T cells. Absolute numbers of circulating $CD8^+$/$CD4^+$ T cells and $CD11b^+$ cells (control) were determined in mice treated or not with FTY720 in Figure 6C to verify its activity. Shown average and S.D.

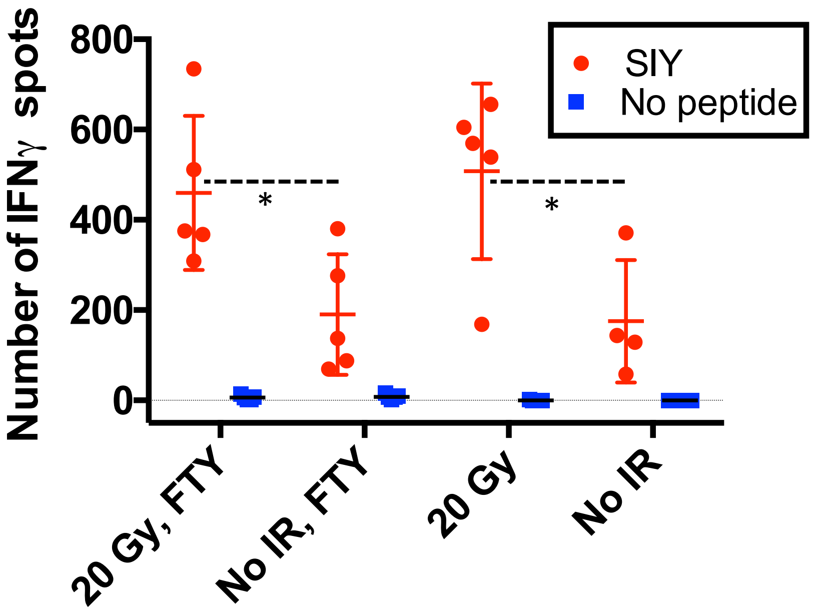

Supplementary Fig. 16. IFNγ production by T cells from irradiated tumors in mice treated or not with FTY720. T cells were purified from irradiated Panc02SIYCerulean tumors 9 days after 20 Gy local IR, and their IFNγ production was analyzed by ELISPOT. Mice were treated or not with FTY720 every 24 h starting 24 h before IR to prevent new T-cell infiltration. Data are from one experiment out of two performed with similar results. Each dot is an individual mouse; shown average and S.D. *, P<0.05. **, P<0.01 (unpaired t-test).

Supplementary Fig. 17

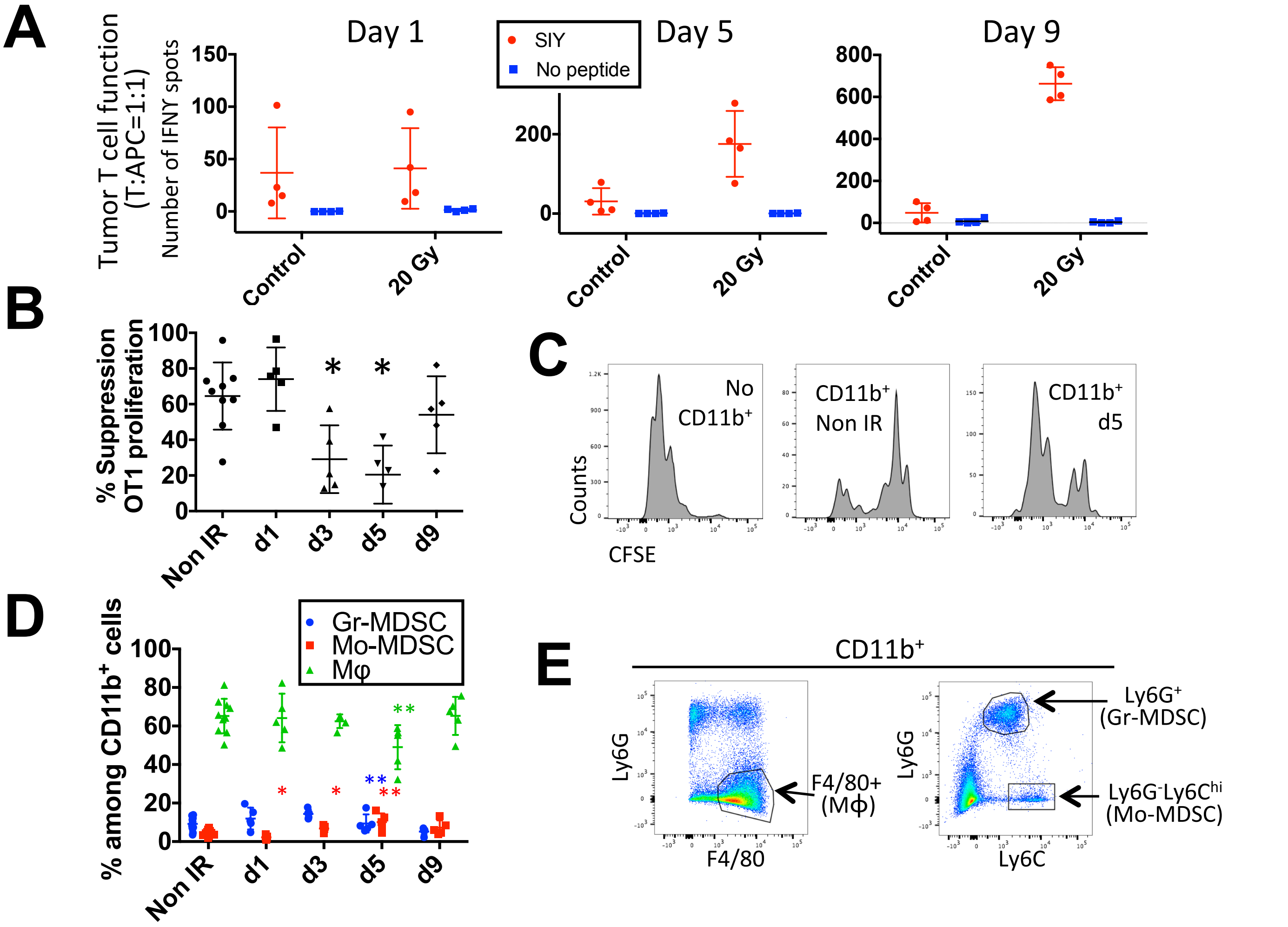

Supplementary Fig. 17. Changes in T cell and myeloid cell function and phenotype in tumors from mice receiving high dose local IR and FTY720 (related to Fig. 6C). Established (3 week) Panc02SIYCerulean tumor-bearing mice were treated or not with 20 Gy local IR. All mice received FTY720 via oral gavage every 24 h starting 24 h before IR until completion of the experiment to prevent new T cell infiltration. **A.** Production of IFNγ by T cells purified from irradiated tumors at days 1, 5 and 9 after IR, measured by ELISPOT. Each dot is an individual mouse. **B.** CD11b^{+} cells were purified from tumors treated as in A, and incubated with CFSE-labeled OT1 splenocytes and OVA peptide to test their ability to suppress T cell proliferation at different times after IR. N=5 mice per group except Non IR (N=9). **C.** Representative FACS plots showing CFSE dilution by OT1 cells in control wells (no CD11b^{+} added) or wells with CD11b^{+} cells purified from untreated mice (Non IR) or mice treated with 20 Gy , at day 5 after IR (d5). **D.** Distribution of CD11b^{+} cells from B in myeloid cell subsets. **E.** Gating strategy used for D. B-E: Two experiments were performed, one comparing tumors that had been irradiated 1 or 3 days before the experiment, vs. non-irradiated tumors; in a second experiment, mice received IR 5 or 9 days before sacrifice, or were not irradiated. In B and D, control unirradiated mice from the two experiments were pooled ("Non IR") and each subset and time point was compared to pooled Non IR. Shown average and S.D; P values by unpaired t-test. *, P<0.05. **, P<0.01.

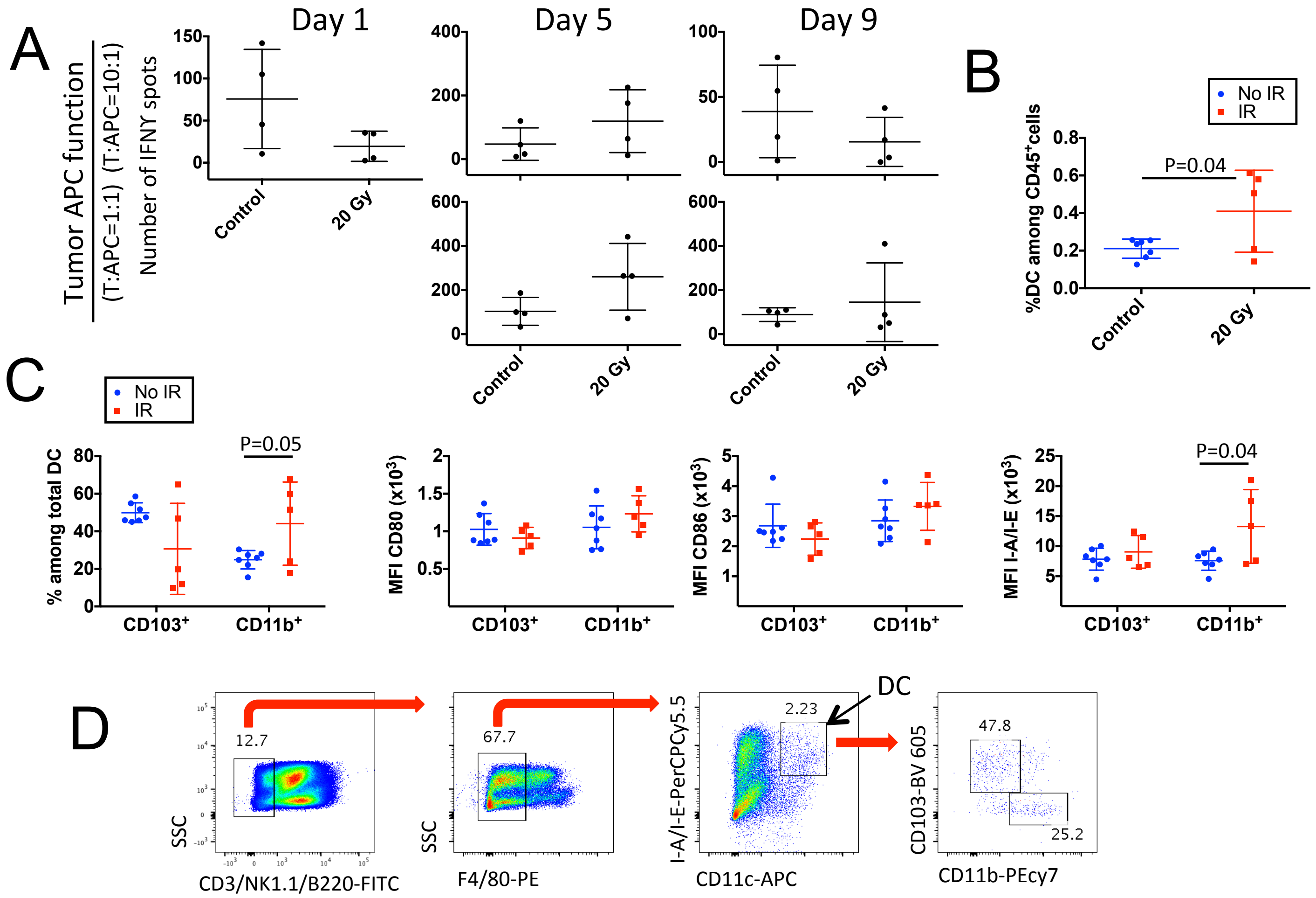

Supplementary Fig. 18. Changes in DC numbers and phenotype in tumors from mice receiving local high dose IR and FTY720 treatment to prevent new T cell infiltration (related to Fig 6C). A. Production of IFNγ by 2C T cells stimulated with CD11c⁺ cells (APC) purified from the same tumors used in Suppl. Fig. 17. Each dot represents an individual mouse. **B-D.** Changes in DC representation and phenotype were analyzed in CD45⁺ cells magnetically purified from tumors at day 5 after IR. Each dot represents an individual mouse. **B.** % CD11c⁺MHC-II⁺ (DC) cells in total CD45⁺ cells, and **C.** Distribution of DC in CD103⁺/CD11b⁺ subsets and levels of expression of DC activation markers on each subset in unirradiated vs. irradiated tumors. **D.** Gating strategy used to defineCD103⁺ and CD11b⁺ DC subsets. Shown average and S.D; P values by unpaired t-test.

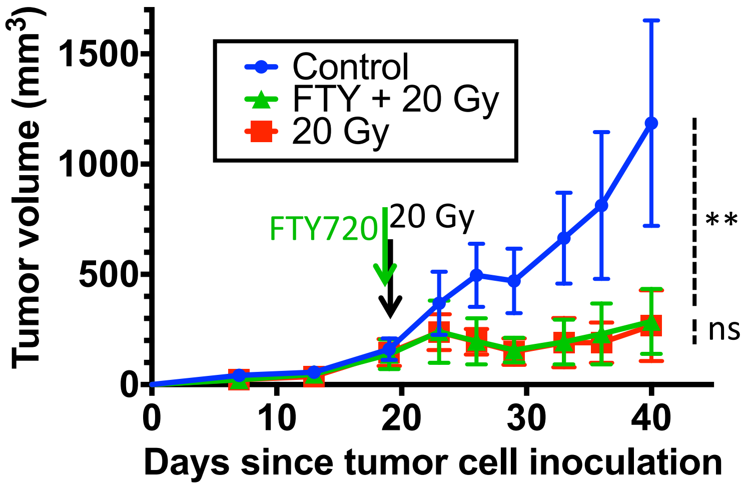

Supplementary Fig. 19. Intratumoral preexistent T cells suffice to control Panc02SIYCerulean tumors without the contribution of newly infiltrating T cells. B6 mice bearing Panc02SIYCerulean s.c. tumors were treated at day 19 after tumor-cell inoculation with 20 Gy local IR. Some mice also received 20 µg FTY720 (FTY) via oral gavage every 24 h, starting 3 h before IR and until day 40. N=8 mice per group except control (N=5). Shown average and S.D. **, P<0.01 (unpaired t-test with Welch's correction at day 40).

Supplementary Table 1. Statistical significance of differences in the effect of IR (fold decrease) in the number of parenchymal CD8$^+$ T cells/g across different tissues.

	P value summary	Geometric mean	Confidence interval	t value	df	P value
LN vs. Spleen	ns	0.9544	0.2873 to 3.171	0.1079	4	0.9193
LN vs. Liver	***	0.07793	0.03531 to 0.1720	8.286	5	0.0004
LN vs. IEL	**	0.02870	0.004797 to 0.1717	5.102	5	0.004
LN vs. Tumor	***	0.01498	0.003485 to 0.06440	7.405	5	0.0007
Spleen vs. Liver	***	0.08383	0.04636 to 0.1516	11.62	4	0.0003
Spleen vs. IEL	*	0.04232	0.005036 to 0.3557	4.125	4	0.0146
Spleen vs. Tumor	***	0.02192	0.006777 to 0.07087	2.405	4	0.0008
Liver vs. IEL	ns	0.3683	0.06025 to 2.251	1.419	5	0.2152
Liver vs. Tumor	*	0.1922	0.04673 to 0.7906	2.998	5	0.0302
IEL vs. Tumor	ns	0.5220	0.1374 to 1.983	1.252	5	0.2660

df, degrees of freedom; ns, not significant. Related to Figure 3C.

Supplementary Table 2. **GSEA Hallmark signatures enriched in tumor vs. LN T cells.**

Name	Enrichment Score	FDR q-val
Hallmark_angiogenesis	0.8134586	0
Hallmark_epithelial_mesenchymal_transition	0.7779522	0
Hallmark_cholesterol_homeostasis	0.76271915	0
Hallmark_hypoxia	0.71806777	0
Hallmark_tnfa_signaling_via_nfkb	0.70413136	0
Hallmark_coagulation	0.6979829	0
Hallmark_protein_secretion	0.6804933	0
Hallmark_uv_response_dn	0.67722726	0
Hallmark_complement	0.6621574	0
Hallmark_il6_jak_stat3_signaling	0.6590407	0
Hallmark_apoptosis	0.6571919	0
Hallmark_tgf_beta_signaling	0.6507696	4.88E-04
Hallmark_inflammatory_response	0.6401307	0
Hallmark_kras_signaling_up	0.63761026	0
Hallmark_glycolysis	0.60729474	0
Hallmark_p53_pathway	0.6036037	0
Hallmark_mtorc1_signaling	0.58386016	0
Hallmark_reactive_oxigen_species_pathway	0.5772216	0.0080058
Hallmark_adipogenesis	0.5755323	6.10E-05
Hallmark_estrogen_response_late	0.55888486	2.73E-04
Hallmark_il2_stat5_signaling	0.55768514	3.01E-04
Hallmark_myogenesis	0.55357033	2.88E-04
Hallmark_apical_surface	0.55173165	0.0150894
Hallmark_androgen_response	0.5408939	0.0036154
Hallmark_estrogen_response_early	0.5343756	0.0010496
Hallmark_apical_junction	0.53242785	6.82E-04
Hallmark_peroxisome	0.5232168	0.0047586
Hallmark_allograft_rejection	0.5225824	0.0014078
Hallmark_notch_signaling	0.5181071	0.0783368
Hallmark_hedgehog_signaling	0.5054939	0.0876418
Hallmark_interferon_gamma_response	0.49924973	0.0034960
Hallmark_mitotic_spindle	0.48338783	0.0057354
Hallmark_pancreas_beta_cells	0.47784835	0.1291808
Hallmark_heme_metabolism	0.43603775	0.0294758
Hallmark_unfolded_protein_response	0.41791424	0.0860558
Hallmark_uv_response_up	0.41009387	0.0774233
Hallmark_xenobiotic_metabolism	0.4094393	0.0601320
Hallmark_bile_acid_metabolism	0.37937248	0.2024376
Hallmark_interferon_alpha_response	0.32377967	0.5406943
Hallmark_pi3k_akt_mtor_signaling	0.31798315	0.5168584

Shown 40 top Hallmark signatures. Related to Figure 4.
