# Supplementary material for: Tumor-reprogrammed resident T cells resist radiation to control tumors
Source: Nat Commun. 2019 Sep 2;10:3959. doi: 10.1038/s41467-019-11906-2 (PMC6718618; doi:10.1038/s41467-019-11906-2)
Supplement: Supplementary file 6 — Reporting Summary [file 41467_2019_11906_MOESM6_ESM.pdf]

nature research

Corresponding author(s): Ainhoa Arina

Last updated by author(s): May 31, 2019

Reporting Summary

Nature Research wishes to improve the reproducibility of the work that we publish. This form provides structure for consistency and transparency in reporting. For further information on Nature Research policies, see Authors & Referees and the Editorial Policy Checklist.

Statistics

For all statistical analyses, confirm that the following items are present in the figure legend, table legend, main text, or Methods section.

n/a	Confirmed	
☐	☒	The exact sample size (*n*) for each experimental group/condition, given as a discrete number and unit of measurement
☐	☒	A statement on whether measurements were taken from distinct samples or whether the same sample was measured repeatedly
☐	☒	The statistical test(s) used AND whether they are one- or two-sided<br>*Only common tests should be described solely by name; describe more complex techniques in the Methods section.*
☒	☐	A description of all covariates tested
☐	☒	A description of any assumptions or corrections, such as tests of normality and adjustment for multiple comparisons
☐	☒	A full description of the statistical parameters including central tendency (e.g. means) or other basic estimates (e.g. regression coefficient) AND variation (e.g. standard deviation) or associated estimates of uncertainty (e.g. confidence intervals)
☐	☒	For null hypothesis testing, the test statistic (e.g. *F*, *t*, *r*) with confidence intervals, effect sizes, degrees of freedom and *P* value noted<br>*Give P values as exact values whenever suitable.*
☒	☐	For Bayesian analysis, information on the choice of priors and Markov chain Monte Carlo settings
☒	☐	For hierarchical and complex designs, identification of the appropriate level for tests and full reporting of outcomes
☒	☐	Estimates of effect sizes (e.g. Cohen's *d*, Pearson's *r*), indicating how they were calculated

Our web collection on statistics for biologists contains articles on many of the points above.

Software and code

Policy information about availability of computer code

Data collection	Leica LAS-AF, FACS DiVa
Data analysis	R (version 3.2.2) , GraphPad Prism 6.0. for Mac, Fiji , SAM

For manuscripts utilizing custom algorithms or software that are central to the research but not yet described in published literature, software must be made available to editors/reviewers. We strongly encourage code deposition in a community repository (e.g. GitHub). See the Nature Research guidelines for submitting code & software for further information.

Data

Policy information about availability of data

All manuscripts must include a data availability statement. This statement should provide the following information, where applicable:
- Accession codes, unique identifiers, or web links for publicly available datasets
- A list of figures that have associated raw data
- A description of any restrictions on data availability

The raw images generated during and/or analysed during the current study for Figures 1 and 2 and Sup. Figure 4 are available from the corresponding author on reasonable request. Gene array accession codes are provided. All other data supporting the findings of this study are available within the paper (and its supplementary information files).

Field-specific reporting

Please select the one below that is the best fit for your research. If you are not sure, read the appropriate sections before making your selection.

☒ Life sciences ☐ Behavioural & social sciences ☐ Ecological, evolutionary & environmental sciences

For a reference copy of the document with all sections, see nature.com/documents/nr-reporting-summary-flat.pdf

Life sciences study design

All studies must disclose on these points even when the disclosure is negative.

Sample size: Sample sizes were chosen based on (i) statistical analysis of initial (pilot) small experiments, performing enough repeats to reach statistical significance and/or (ii) limits imposed by the technical approach used (e.g. longitudinal in vivo imaging experiments, where no more than 2-3 animals can be monitored longitudinally in the same experiment because imaging of each animal takes several hours)

Data exclusions: No data exclusions

Replication: Experiments were independently performed at least twice or as many times as required to reach statistical significance, using the same experimental parameters each time (i.e. procedures, reagents, instrument settings etc)

Randomization: Random allocation when possible (see also Blinding.)

Blinding: One investigator assigned groups of animals to be treated in each group, using random group allocation when possible (i.e. when tumor sizes were equivalent between mouse cages , cages were assigned to groups randomly), and also assigned treatment codes to each group. When tumor size average varied between cages at the time of treatment, mice from different cages were assigned to groups to make the average size equivalent among groups at the time of treatment. Whenever possible, a different investigator monitored tumor size during the rest of the experiment.

Reporting for specific materials, systems and methods

We require information from authors about some types of materials, experimental systems and methods used in many studies. Here, indicate whether each material, system or method listed is relevant to your study. If you are not sure if a list item applies to your research, read the appropriate section before selecting a response.

Materials & experimental systems

n/a	Involved in the study	
☐	☒	Antibodies
☐	☒	Eukaryotic cell lines
☐	☐	Palaeontology
☐	☒	Animals and other organisms
☐	☐	Human research participants
☐	☐	Clinical data

Methods

n/a	Involved in the study	
☐	☐	ChIP-seq
☐	☒	Flow cytometry
☐	☐	MRI-based neuroimaging

Antibodies

Antibodies used: See below

Validation:

Antibody target Supplier Cat. No. Clone Lot No. Validation
CD8α-APC BioLegend 100712 53-6.72 B207080 Mouse splenocytes/PBL
CD8β-PerCPCy5.5 BioLegend 126609 YTS156.7.7 B175736 Mouse splenocytes/PBL
CD4-eFluor780 eBioscience 47-0042-82 RM4-5 4278618 Mouse splenocytes/PBL
CD3-BV711 BioLegend 100241 17A2 B220472 Mouse splenocytes/PBL
CD44-PEcy7 BioLegend 103030 IM7 B193069 Mouse splenocytes/PBL
CD62L-APC BioLegend 104412 MEL-14 B223862 Mouse splenocytes/PBL
α1-APC BioLegend 142605 HMα1 B208503 Data in manuscript
α9 purified R&D Systems AF3827 Polyclonal YLI0315101 Data in manuscript
Anti-Goat IgG-AF594 Life Technologies A11058 Polyclonal 1180089 Data in manuscript
αV-biotin BioLegend 104103 RMV-7 B210818 Data in manuscript
αM /CD11b- PerCPCy5.5 BioLegend 101228 M1/70 B174517 Mouse splenocytes/PBL
CD11b- eFluor780 eBioscience 47-0112-82 M1/70 4277883 Mouse splenocytes/PBL
F4/80-Pac Blue BioLegend 123124 BM8 B205037 Data in manuscript
Ly6C-FITC BioLegend 128006 HK 1.4 B217035 Mouse splenocytes/PBL
Ly6G-PE BioLegend 127648 1A8 B227525 Mouse splenocytes/PBL
CD69-PEcy7 Biolegend 104512 H1.2F3 B180852 Data in manuscript

LFA1-PE Biolegend 141005 B273452 Data in manuscript
FoxP3 PEcy7 eBioscience 25-5773-82 4292088 Data in manuscript
LAG3 biotin Biolegend 125205 B182150 Data in manuscript
TIGIT PE eBioscience 12-9501-82 4301334 Data in manuscript
CD137 APC Biolegend 106110 B227022 Data in manuscript
NK1.1 FITC BD Pharmingen 553164 80219 Mouse splenocytes/PBL
B220 FITC Biolegend 103206 B209103 Mouse splenocytes/PBL
F4/80 PE Biolegend 123110 B223150 Data in manuscript
CD11c APC Biolegend 117310 B262130 Data in manuscript
I-A/I-E PerCPCy5.5 Biolegend 107626 B253463 Data in manuscript
Thy1.1 PerCPCy5.5 Biolegend 202512 B202057 Mouse splenocytes/PBL
Thy1.2 Pacific Blue Biolegend 140306 B245912 Mouse splenocytes/PBL
CD80 Pacific Blue Biolegend 104724 B258827 Data in manuscript
CD86 biotin Biolegend 105003 B245380 Data in manuscript
CD103-PE Biolegend 121429 2E7 B219931 Data in manuscript
CD103-PEDazzle594 Biolegend 121430 B261760 Data in manuscript
TGFβ
(in vivo) BioXcel BE0057 1D11.16.8 642917J1 Chen et al. PLoS One, 2014 Jan 9;9(1):e85398.
PD-L1
(in vivo) BioXcel BE0101 10F.9G2 665717O1 Deng et al, J Clin Invest. 2014 Feb;124(2):687-95.
CD8
(in vivo) BioXcel BE0004-1 53-6.72 634316D1 Deng et al, J Clin Invest. 2014 Feb;124(2):687-95.

Eukaryotic cell lines

Policy information about cell lines

Cell line source(s)

Panc02 murine pancreatic adenocarcinoma cells donated by Michael Gough (Oregon Health and Science University) were retrovirally infected with pMFG (SIY)3-Cerulean provided by Dr. Hans Schreiber, as described (ref. 22). After infection, cells were FACS-sorted for high expression of SIY-Cerulean to generate the Panc02-SIY-Cerulean cell line. MC38 colon adenocarcinoma cell line was provided by Dr. Yang-Xin Fu. MC38-Cerulean cells have been described (ref. 23) and were donated by Dr. Hans Schreiber.

Authentication

No authentication performed

Mycoplasma contamination

All cell lines tested negative for Mycoplasma contamination by PCR, in house and by out-sourced tests (Idexx BioAnalytics)

Commonly misidentified lines
(See ICLAC register)

No commonly misidentified cell lines were used

Palaeontology

Specimen provenance

Provide provenance information for specimens and describe permits that were obtained for the work (including the name of the issuing authority, the date of issue, and any identifying information).

Specimen deposition

Indicate where the specimens have been deposited to permit free access by other researchers.

Dating methods

If new dates are provided, describe how they were obtained (e.g. collection, storage, sample pretreatment and measurement), where they were obtained (i.e. lab name), the calibration program and the protocol for quality assurance OR state that no new dates are provided.

☐ Tick this box to confirm that the raw and calibrated dates are available in the paper or in Supplementary Information.

Animals and other organisms

Policy information about studies involving animals; ARRIVE guidelines recommended for reporting animal research

Laboratory animals

T cell reporter mice were generated by crossing CD4-cre (JAX 017336) or Lck-cre (JAX 003802) with R26R-EYFP (JAX 006148) mice, all from Jackson. OT1Rag-/- mice were kindly donated by Dr. Hans Schreiber (University of Chicago). T cell reporter and OT1Rag-/- males and females were used between 6 and 20 weeks. 6-8 week old C57BL/6 female mice were purchased from Jackson or Harlan.

Wild animals

Provide details on animals observed in or captured in the field; report species, sex and age where possible. Describe how animals were caught and transported and what happened to captive animals after the study (if killed, explain why and describe method; if released, say where and when) OR state that the study did not involve wild animals.

Field-collected samples

For laboratory work with field-collected samples, describe all relevant parameters such as housing, maintenance, temperature, photoperiod and end-of-experiment protocol OR state that the study did not involve samples collected from the field.

Ethics oversight

Animal care and use were in accordance with institutional and NIH protocols and guidelines. All studies were approved by the Animal Care and Use Committee of The University of Chicago.

Note that full information on the approval of the study protocol must also be provided in the manuscript.

Human research participants

Policy information about studies involving human research participants

Population characteristics	*Describe the covariate-relevant population characteristics of the human research participants (e.g. age, gender, genotypic information, past and current diagnosis and treatment categories). If you filled out the behavioural & social sciences study design questions and have nothing to add here, write "See above."*
Recruitment	*Describe how participants were recruited. Outline any potential self-selection bias or other biases that may be present and how these are likely to impact results.*
Ethics oversight	*Identify the organization(s) that approved the study protocol.*

Note that full information on the approval of the study protocol must also be provided in the manuscript.

Clinical data

Policy information about clinical studies

All manuscripts should comply with the ICMJE guidelines for publication of clinical research and a completed CONSORT checklist must be included with all submissions.

Clinical trial registration	*Provide the trial registration number from ClinicalTrials.gov or an equivalent agency.*
Study protocol	*Note where the full trial protocol can be accessed OR if not available, explain why.*
Data collection	*Describe the settings and locales of data collection, noting the time periods of recruitment and data collection.*
Outcomes	*Describe how you pre-defined primary and secondary outcome measures and how you assessed these measures.*

ChIP-seq

Data deposition

- [ ] Confirm that both raw and final processed data have been deposited in a public database such as GEO.
- [ ] Confirm that you have deposited or provided access to graph files (e.g. BED files) for the called peaks.

Data access links<br>*May remain private before publication.*	*For "Initial submission" or "Revised version" documents, provide reviewer access links. For your "Final submission" document, provide a link to the deposited data.*
Files in database submission	*Provide a list of all files available in the database submission.*
Genome browser session<br>(e.g. UCSC)	*Provide a link to an anonymized genome browser session for "Initial submission" and "Revised version" documents only, to enable peer review. Write "no longer applicable" for "Final submission" documents.*

Methodology

Replicates	*Describe the experimental replicates, specifying number, type and replicate agreement.*
Sequencing depth	*Describe the sequencing depth for each experiment, providing the total number of reads, uniquely mapped reads, length of reads and whether they were paired- or single-end.*
Antibodies	*Describe the antibodies used for the ChIP-seq experiments; as applicable, provide supplier name, catalog number, clone name, and lot number.*
Peak calling parameters	*Specify the command line program and parameters used for read mapping and peak calling, including the ChIP, control and index files used.*
Data quality	*Describe the methods used to ensure data quality in full detail, including how many peaks are at FDR 5% and above 5-fold enrichment.*
Software	*Describe the software used to collect and analyze the ChIP-seq data. For custom code that has been deposited into a community repository, provide accession details.*

Flow Cytometry

Plots

Confirm that:

☒ The axis labels state the marker and fluorochrome used (e.g. CD4-FITC).

☒ The axis scales are clearly visible. Include numbers along axes only for bottom left plot of group (a 'group' is an analysis of identical markers).

☒ All plots are contour plots with outliers or pseudocolor plots.

☒ A numerical value for number of cells or percentage (with statistics) is provided.

Methodology

Sample preparation	Tumors were excised and digested for 20-45 minutes at 37C with 75 µg/mL liberase TM (Roche) and 20 µg/mL DNAse I (Sigma). For studies on tissue resident T cells, resident (non-circulating) CD8+ T cells were distinguished from circulating T cells trapped in the organ vasculature using the intravascular staining technique. Briefly, mice were injected with 3 µg of anti-CD8α-APC antibody i.v. 3-5 minutes before sacrifice. Organs were isolated after perfusion with PBS with 75 U/mL heparin. Spleens, lymph nodes and livers were mechanically disrupted through a sterile 70-µm nylon mesh filter. Hepatocytes were excluded from the liver samples by performing a single-step 35% Percoll centrifugation (GE Healthcare, Chalfont St Giles, UK) and using only the pelleted cells. Lungs were diced into 1 mm pieces, digested with 125 µg/mL liberase TM and 20 µg/mL DNAse I for 20 min at 37C, passed through a sterile 70-µm nylon mesh filter and spun down in 40% Percoll (adapted from 35). Erythrocytes from spleens, lungs and livers were lysed with ACK buffer. For extraction of IEL, small intestines were cut into 1 cm long pieces after removal of Peyer's patches, and fragments were incubated for 20 min at 37C under gentle shaking in complete RPMI media (10% FBS, HEPES, Non-essential aminoacids, L-Glutamine, 2-mercaptoethanol) with 5 mM EDTA and 0.145 mg/mL DTT. Fragments of intestine were then filtered using a kitchen strainer and further stripped off the epithelium containing the IELs through subsequent rounds of vigorous shaking in a 50 mL conical tube with wash buffer (RPMI supplemented with HEPES and Penicillin/Streptomycin), followed by filtering and centrifugation of the flow through. Single-cell suspensions from all organs were stained with relevant antibodies for 15 min at 4°C, and washed twice with cold PBS. Blood samples were stained, treated with NH_4Cl red blood cell lysis buffer and immediately acquired (without washing) after addition of BrightCount beads (Invitrogen) to determine the absolute counts of cell populations in PBL. For sorting of CD8+CD44+CD62L- T cells from tumors, cell suspensions obtained by enzymatic digestion were spun down in 35% Percoll and enriched for CD8+ T cells using EasySep CD8+ positive selection kit before staining with appropriate antibodies and FACS-sorting. Samples were typically incubated with antibodies for 10-15 minutes at 4C and washed twice with PBS.
Instrument	Data were acquired on a LSRII or LSR-Fortessa (BD)
Software	Data were collected using FACS DiVa (BD) and analyzed using FlowJo software
Cell population abundance	Purity of sorted fractions is typically around 99.5%
Gating strategy	In Sup. Fig 3, preexistent T cells were gated as Thy1.2+, whereas newly infiltrating T cells were Thy1.1+. Suppl. Fig. 5. provides the gating strategy fused to determine percentages of circulating and intratumor T cells in T cell reporter mice receiving WBI (related to Figures 3A and 3B). For the analysis of tissue-resident memory cells (Fig.3C-F) , live cells were gated on FSC/SSC and then CD8betaPerCPCy5.5+CD8alphaAPC- cells were gated as parenchymal CD8 T cells (gating strategy in Sup. Fig 6A); parenchymal T cells were analyzed for expression of tissue-resident or memory markers. For Figure 4, CD44+CD62L- CD8+ T cells were gated, as shown in Suppl. Figure 8. For Sup. Fig 12, CD3+CD4+ and CD3+CD8+ T cells within the live cell gate to analyze integrin subunit expression. For Sup. Fig 17D, live cells were gated on FSC/SSC, then CD11b+ cells were gated on a SSC/CD11b plot. The gates considered for subpopulation analysis are provided on Sup. Fig. 17E. Gating strategy for DC analysis is on Suppl. Figure 18.

☒ Tick this box to confirm that a figure exemplifying the gating strategy is provided in the Supplementary Information.

Magnetic resonance imaging

Experimental design

Design type	*Indicate task or resting state; event-related or block design.*
Design specifications	*Specify the number of blocks, trials or experimental units per session and/or subject, and specify the length of each trial or block (if trials are blocked) and interval between trials.*
Behavioral performance measures	*State number and/or type of variables recorded (e.g. correct button press, response time) and what statistics were used to establish that the subjects were performing the task as expected (e.g. mean, range, and/or standard deviation across subjects).*

Acquisition

Imaging type(s) | *Specify: functional, structural, diffusion, perfusion.*

Field strength | *Specify in Tesla*

Sequence & imaging parameters | *Specify the pulse sequence type (gradient echo, spin echo, etc.), imaging type (EPI, spiral, etc.), field of view, matrix size, slice thickness, orientation and TE/TR/flip angle.*

Area of acquisition | *State whether a whole brain scan was used OR define the area of acquisition, describing how the region was determined.*

Diffusion MRI ☐ Used ☐ Not used

Preprocessing

Preprocessing software | *Provide detail on software version and revision number and on specific parameters (model/functions, brain extraction, segmentation, smoothing kernel size, etc.).*

Normalization | *If data were normalized/standardized, describe the approach(es): specify linear or non-linear and define image types used for transformation OR indicate that data were not normalized and explain rationale for lack of normalization.*

Normalization template | *Describe the template used for normalization/transformation, specifying subject space or group standardized space (e.g. original Talairach, MNI305, ICBM152) OR indicate that the data were not normalized.*

Noise and artifact removal | *Describe your procedure(s) for artifact and structured noise removal, specifying motion parameters, tissue signals and physiological signals (heart rate, respiration).*

Volume censoring | *Define your software and/or method and criteria for volume censoring, and state the extent of such censoring.*

Statistical modeling & inference

Model type and settings | *Specify type (mass univariate, multivariate, RSA, predictive, etc.) and describe essential details of the model at the first and second levels (e.g. fixed, random or mixed effects; drift or auto-correlation).*

Effect(s) tested | *Define precise effect in terms of the task or stimulus conditions instead of psychological concepts and indicate whether ANOVA or factorial designs were used.*

Specify type of analysis: ☐ Whole brain ☐ ROI-based ☐ Both

Statistic type for inference
(See Eklund et al. 2016) | *Specify voxel-wise or cluster-wise and report all relevant parameters for cluster-wise methods.*

Correction | *Describe the type of correction and how it is obtained for multiple comparisons (e.g. FWE, FDR, permutation or Monte Carlo).*

Models & analysis

n/a	Involved in the study
☐	☐ Functional and/or effective connectivity
☐	☐ Graph analysis
☐	☐ Multivariate modeling or predictive analysis

Functional and/or effective connectivity | *Report the measures of dependence used and the model details (e.g. Pearson correlation, partial correlation, mutual information).*

Graph analysis | *Report the dependent variable and connectivity measure, specifying weighted graph or binarized graph, subject- or group-level, and the global and/or node summaries used (e.g. clustering coefficient, efficiency, etc.).*

Multivariate modeling and predictive analysis | *Specify independent variables, features extraction and dimension reduction, model, training and evaluation metrics.*
